# Supplementary material for: Microparticle traction force microscopy reveals subcellular force exertion patterns in immune cell–target interactions
Source: Nat Commun. 2020 Jan 7;11:20. doi: 10.1038/s41467-019-13804-z (PMC6946705; doi:10.1038/s41467-019-13804-z)
Supplement: Supplementary file 1 — Supplementary Information [file 41467_2019_13804_MOESM1_ESM.pdf]

SUPPLEMENTARY INFORMATION

Microparticle traction force microscopy reveals subcellular force exertion patterns in immune cell-target interactions

Vorselen *et al.*

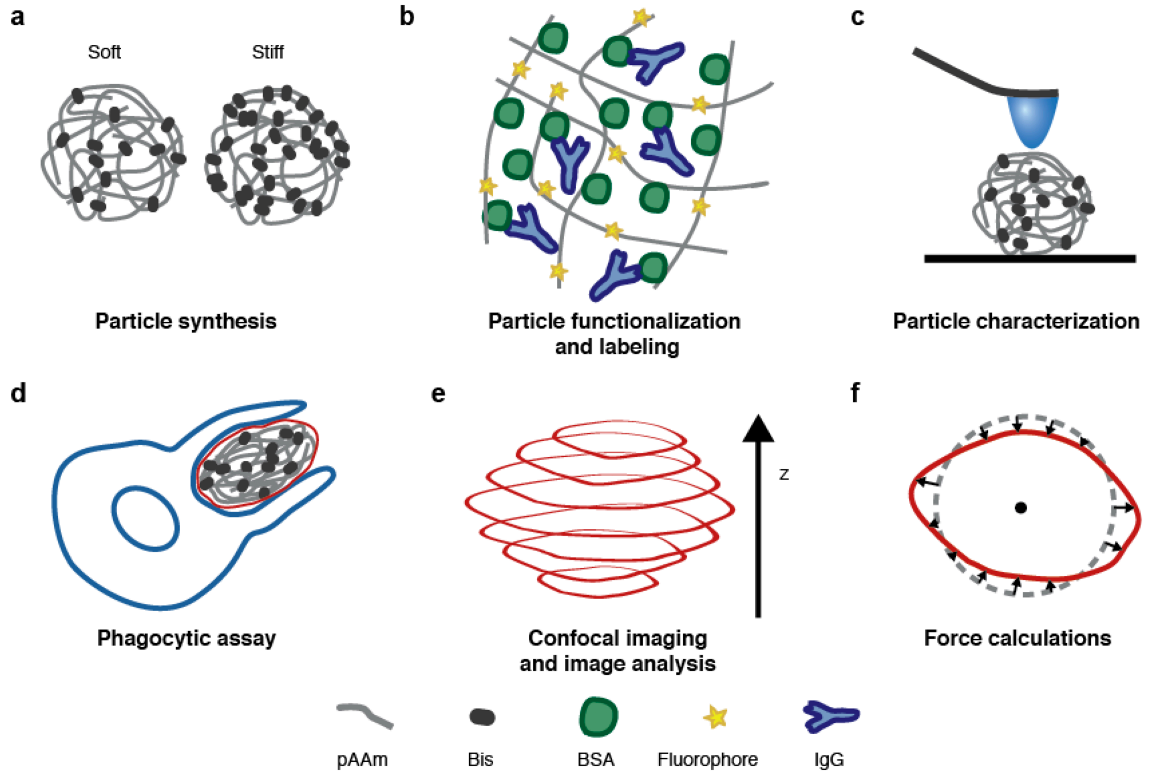

Supplementary Figure 1: Schematic diagram of the developed method. a, Rigidity of spherical deformable polyacrylamide-co-acrylic acid microparticles (DAAM-particles) can be tuned by adjusting the crosslinker (bisacrylamide) or total acrylamide concentration. **b,** Inclusion of acrylic acid (AAc) in the DAAM-particle allows direct conjugation of proteins and carboxyl-reactive fluorescent dyes to the gel. In our approach, the gel is functionalized further with αBSA-IgG. **c**, The mechanical properties of DAAM-particles can be characterized using atomic force microscopy (AFM). Using pyramidal tips with large end radius (> 0.5 μm) allows both imaging and Hertzian indentations of MPs. **d,** Phagocytes (in this manuscript J774 macrophage-like cells) are exposed to DAAM-particles functionalized to trigger phagocytosis. When the DAAM-particles are of sufficiently low rigidity, the forces exerted by the cell during phagocytosis will deform the particles. **e,** Confocal microscopy allows visualization of the particle shape using the dye embedded in the gel. Image analysis allows reconstruction of the particle boundary with superresolution in the radial direction. **f,** As the mechanical properties of the DAAM-particles are known, isotropic and homogeneous, observed deformations can be used to calculate the forces exerted by the cell.

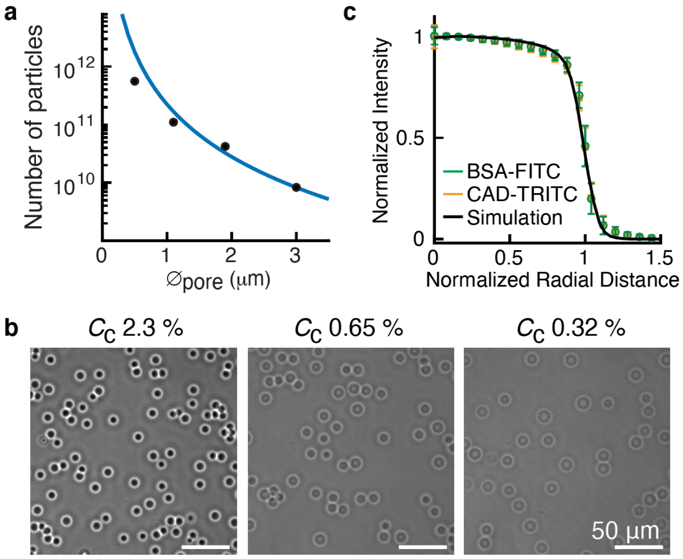

Supplementary Figure 2: Batch production of DAAM-particles using hydrophobic Shirasu Porous Glass (SPG). a, SPG membrane pore size versus estimated number of particles per batch, calculated as V_{aq}/V_{MP}, where V_{aq} is the volume of the aqueous phase containing the gel mixture (10 mL), and V_{MP} is the volume of a single particle, as quantified in figure 1b (main text). The blue line corresponds to the linear relation between SPG membrane pore size and particle size with slope 4.9 (Fig. 1b, main text). Note that this estimate assumes no gel swelling, which results in an underestimate of the number of particles per batch. **b,** Phase images of DAAM-particles that were made with the same pore size membrane and different crosslinker concentrations (C_c). Lowering C_c results in more hydrogel swelling (as evidenced from the reduced contrast indicating lower polymer density) and larger particles. **c,** Radial fluorescence intensity profile illustrating particle radial homogeneity (> 100 particles were used to create this average). Identical to figure 1e (main text), but for C_c 0.32%. Source data are provided as a Source Data file.

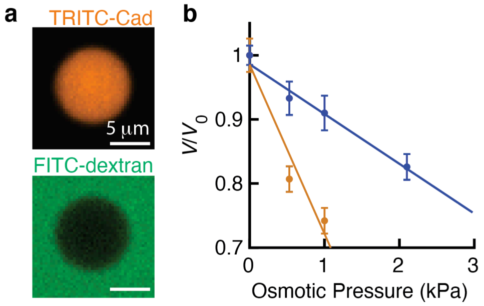

Supplementary Figure 3: Bulk modulus measurement of DAAM-particles. a, Confocal microscopy image of a DAAM-particle (C_c 0.65%) in a 13 mg/mL FITC-dextran solution (Osmotic pressure $\Pi = 0.53$ kPa). The polymer is excluded from the DAAM-particle, which is essential for volume compression of the particle under osmotic pressure. **b,** DAAM-particle volume dependence on osmotic pressure measured in various concentration dextran solutions (13 mg/mL, $\Pi = 0.53$ kPa; 21 mg/mL, $\Pi = 1$ kPa; 33 mg/mL, $\Pi = 2.1$ kPa). Particle volume was estimated from the slice with the maximum surface area for each particle and then normalized by the volume in PBS (V_0). Measurements were only made for particle with crosslinker concentrate C_c 0.65% (orange) and 2.3% (purple) since softer particles would deform far beyond the expected linear elastic regime (strain $\varepsilon > 10\%$), and we lack reliable estimates of the osmotic pressure of low concentration 2,000,000 g/mol dextran solutions[1]. At least 124 particles were measured in each condition. Error bars indicate standard error of the mean (s.e.m.). Solid lines represent linear fits. The bulk modulus (B) of the particles is the reciprocal of the linear fits, which we estimate as 3.8 ± 0.5 kPa for DAAM-particles with C_c 0.65% and as 13 ± 2 kPa for DAAM-particles with C_c 3.2%. Errors on bulk modulus estimates represent standard deviations of repeated bulk modulus derivations using bootstrapped samples. Finally, combining these bulk modulus estimates with the Young's modulus (E_y) estimates obtained by AFM (figure 1j,e in the main text) allows us to estimate the Poisson's ratio of the DAAMPs, which is difficult to measure directly[2], as $\nu = 0.5 - E_y/6B$. The Poisson's ratio obtained was 0.42 ± 0.02 for C_c 3.2% and 0.44 ± 0.01 for C_c 0.65% DAAMPs, where errors were estimated by bootstrap as above. Source data are provided as a Source Data file.

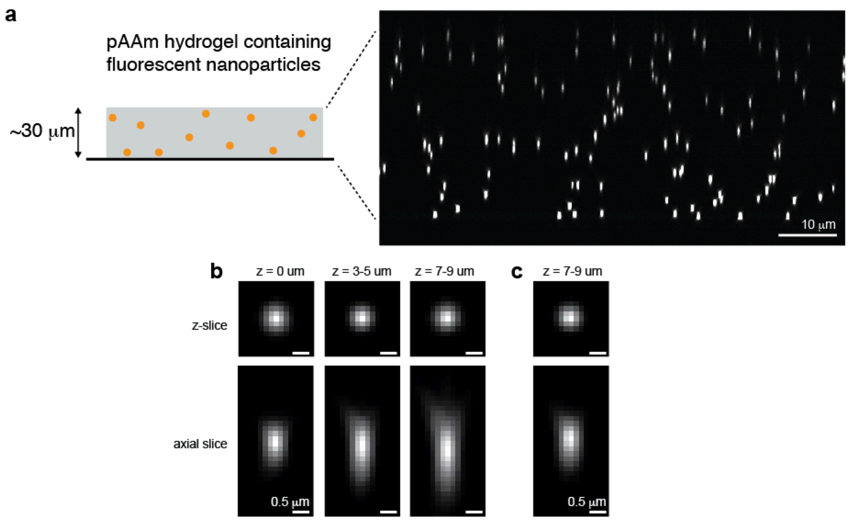

Supplementary Figure 4: Depth-dependent measurement of point-spread-functions (PSFs) in adherent hydrogels. a, (Left) Diagram of the approach: polyacrylamide (pAAm) hydrogels that are covalently bound to the glass surface, and in which fluorescent nanoparticles (~100 nm diameter) were incorporated, were imaged on a confocal microscope. (Right) Confocal image (axial-slice) of such a hydrogel. It can be seen that particles further from the surface appear dimmer and elongated along the axial direction. The location of the glass slide can be determined from the background signal, when the contrast is enhanced (now shown). **b,** Reconstructed point-spread-functions (PSFs) from red 100 nm nanoparticles imaged at various distance from the surface (z). Fluorescent signals of over 50 nanoparticles within each distance interval were superimposed with subpixel resolution and averaged. In z-slices the particles look identical, but in axial-direction the PSF width increases with distance to the surface. The middle PSF was used for simulating the appearance of a homogeneous microsphere on the confocal (Fig. 1c, main text and Supplementary Fig. 2). The PSF on the right was used for deconvolution of DAAM-particles in PBS (Fig. 4, main text). **c,** Similar to **b,** but reconstructed from 100 nm yellow-green nanoparticles measured in high refractive index (RI) medium (Vectashield (VS); RI ~ 1.45). This PSF was used for deconvolution of DAAM-particles imaged in VS (Fig. 3, main text).

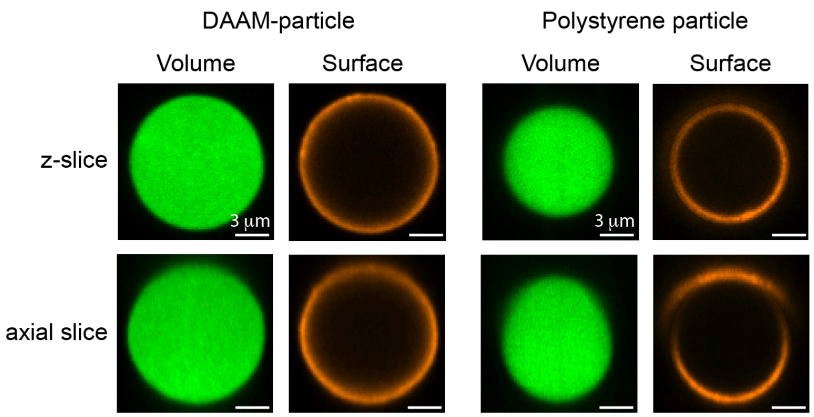

Supplementary Figure 5: Microparticle lensing and image distortion in high refractive index media. *Z*- and axial slices from representative confocal image stacks through a DAAM-particles (C_c 0.32%) and a 10 μm fluorescent PS particle. DAAMPs were functionalized with Alexa 488-cadaverine. Surface of DAAMPs and PS MPs was functionalized with BSA, αBSA-IgG, and Alexa 546-GαR-IgG imaged in Vectashield (refractive index: ~1.45). The polystyrene particle appears deformed (egg-shaped) in the axial direction. The surface stain is also clearly distorted: the signal is displaced from the actual surface, and shows large intensity variations, in the upper hemisphere. These artefacts arise due to the refractive index mismatch between particle and medium, which results in the particle acting as a lens. Such gross artefacts are not noticeable with DAAM-particles, since their refractive index is very close to the medium (Fig. 1d, main text).

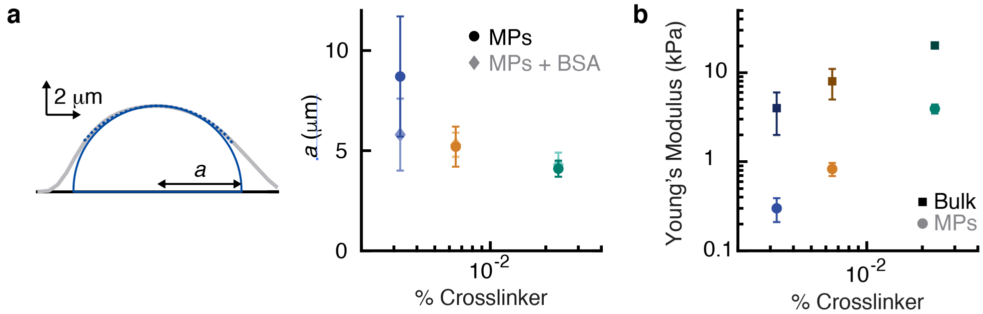

Supplementary Figure 6: Contact radius determination and comparison of elastic properties between DAAM-particles and bulk gels. a, (Left) Shape determination from atomic force microscopy (AFM) images. In grey, a line profile from an image through the maximum of a particle, measured along the slow scanning axis. A spherical arc was fitted to the upper half of the line profile (since the lower part of a sphere cannot be imaged with AFM). The particle radius of curvature (R) was then estimated as $R = R_{tot} - R_{tip}$, where R_{tot} is the radius of the fitted spherical arc, and R_{tip} is the radius of the AFM tip. Under assumption that the particles form spherical caps, and using the height estimated from the image, this yields an estimate of the particle shape and, hence, the contact radius with the surface (a). (Right) Quantification of the particle contact radius with the surface for particles with and without functionalization, and various crosslinker concentration. Error bars indicate standard deviations. **b,** Comparison of Young's moduli of microparticles (~10 µm) with bulk adherent hydrogels of identical composition, *i.e.* same total acrylamide concentration, same crosslinker concentration and same acrylic acid concentration. For the bulk gels, n = 15 indentations in unique locations. Data for MPs is identical to figure 1f in the main text. Error bars mark standard deviations between individual force curves (bulk gels), or standard deviations from particle-to-particle, where for each particle the Young's modulus was estimated as the average from 2-5 indentation curves (MPs). Source data are provided as a Source Data file.

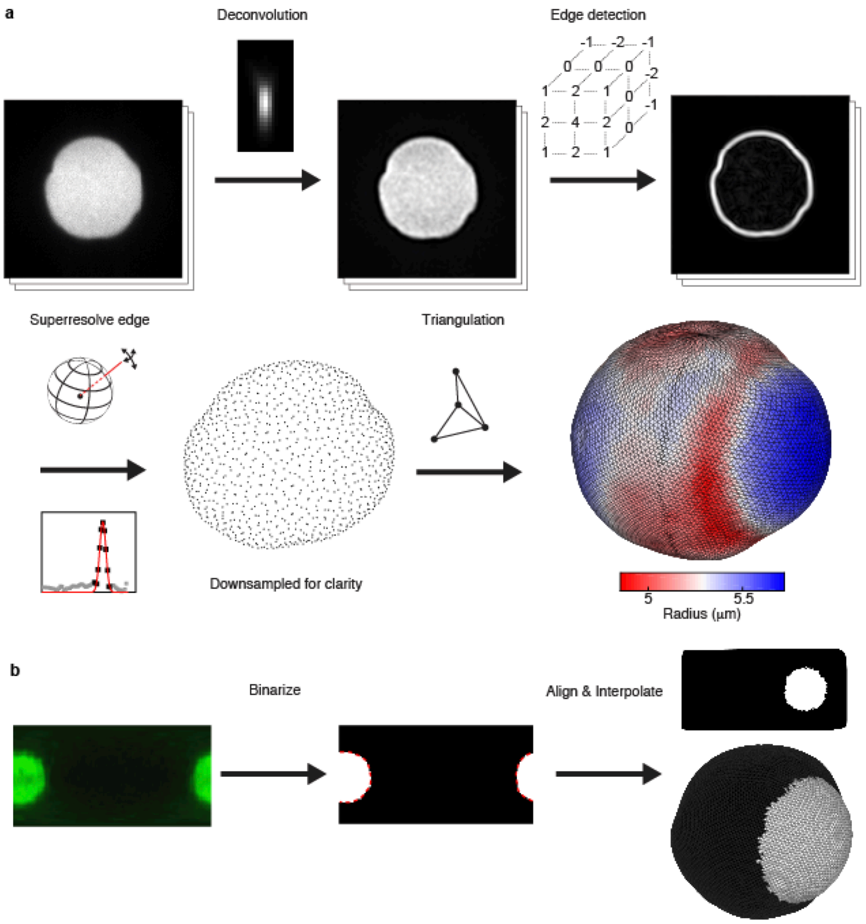

Supplementary Figure 7: Image analysis steps for 3D particle shape reconstruction and free boundary analysis. **a,** Particle shape reconstruction. Confocal image stacks were first deconvolved, using a point spread function measured with 100 nm red fluorescent nanoparticles, imaged ~8 μm from the surface (Supplementary Fig. 3). Subsequently, edge detection was performed using convolution with the 3 kernels of the 3D-Sobel Operator, after which a single 3D image was calculated as the magnitude of the 3 resulting images. Next, lines were drawn outward from the centroid with approximately equally spaced angles, and the fluorescent signal along these lines was approximated by interpolation. A Gaussian function (see supplementary discussion) was fit to the peak in the resulting line profile, allowing determination of surface coordinates. Finally, triangulation was performed to calculate particle and surface properties (*e.g.* centroid, volume, surface area, curvature). **b,** Free boundary analysis. The immunostained exposed part of the particle surface was analysed similar to the particle fluorescent signal, except that 1) Edge detection was not performed; 2) A regular grid was used; 3) The peak of the Gaussian profile was not localized, but instead, the integral of the Gaussian was calculated, which was approximated as $I_{tot} = 1.065 * I_{max} * \text{FWHM}$, where I_{tot} is the total fluorescent intensity under the Gaussian, I_{max} is the maximum fluorescent intensity, and FWHM is the full width half maximum of the Gaussian. I_{tot} was more uniform over the MP surface than the I_{max}. A 2D projection of I_{max} was made (left) and the signal was binarized using a region-based active contour (or snake) algorithm[3] (dashed red line). Finally, the binary mask was used to align the particles, with the base of the cup at longitude -0.5π and latitude 0. The mask was then interpolated to determine for each edge coordinate if it was within the area covered by the cell, or part of the traction-free boundary.

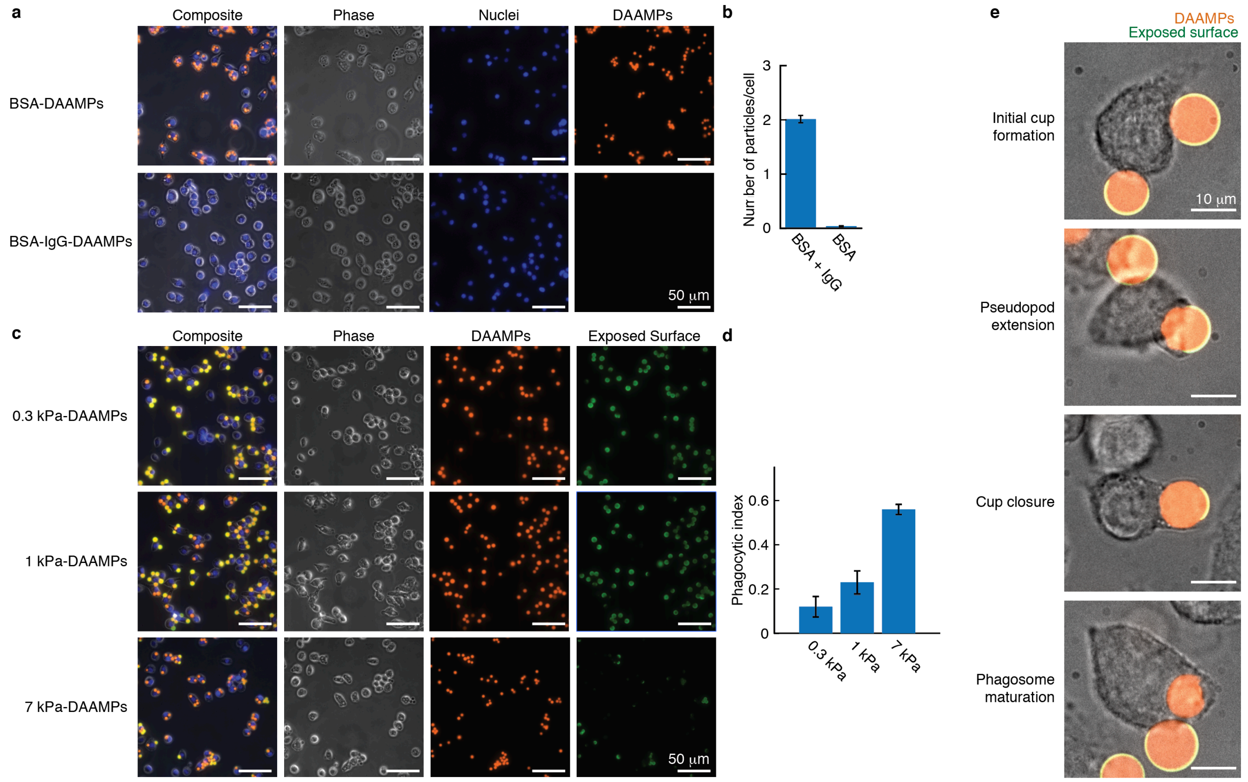

Supplementary Figure 8: Uptake of DAAMPs by J774 macrophage-like cells is ligand and rigidity dependent. **a,** J774 murine macrophage-like cells were seeded in a 12-well plate (1.5 * 10^5 cells/well). The next day, cell nuclei were stained with Hoechst, and then exposed to stiff (crosslinker concentration C_c 2.3%) MPs (~7.5 * 10^5 MPs/well), stained with TRITC-Cadaverine and further functionalized with BSA and αBSA-IgG, or only BSA, for 30 minutes. Cells were then fixed, washed vigorously and imaged (40×). Typical fields of view (FOV) are shown. **b,** Quantification of the number of beads (counted from fluorescent TRITC-CAD signal) per cell (counted from fluorescent Hoechst signal). Approximately 400 cells were analysed in each condition. Error bars represent standard deviations estimated as $\sqrt{\lambda}$, where λ is the average number of beads per cell, hence we make the assumption that the number of DAAMPs per cell follows a Poisson distribution. **c,** Similar experimental setup to **a**, but now cells were exposed to DAAMPs of various crosslinker concentrations (C_c 0.32%, 0.65% or 2.3%). Staining was done similarly to **a,** but in addition exposed surface of DAAMPs was immunostained with 4 µg/mL Alexa Fluor 647 donkey anti-rabbit IgG (ThermoFisher Sci., A31573) in PBS, which allows separation of fully internalized from externalized particles. **d,** Quantification of the phagocytic index, which we define as the number of internalized particles (N_i) divided by the total number of particle ($N_{tot} = N_i + N_o$, where N_o is the number of particles that are adherent or partially internalized). More than 200 beads were analysed in each condition, revealing that stiffer particles get engulfed more efficiently than similar softer particles. Error bars represent standard deviations estimated as $\sqrt{N_i N_o / N_{tot}}$. Hence, we make the assumption that internalization of particles can be described as a Bernoulli process. **e,** Larger FOV images from which the panels of figure 3 in the main text were cropped, here showing the entire cells. Composite of brightfield image with confocal z-slices. DAAM-particles were functionalized with TRITC-Cadaverine (Cad), BSA and anti-BSA IgG. J774 murine cells were exposed to DAAM-particles for 5 minutes and subsequently fixed. After fixation, the freely exposed particle surface was immunostained with Alexa-647 secondary antibody.

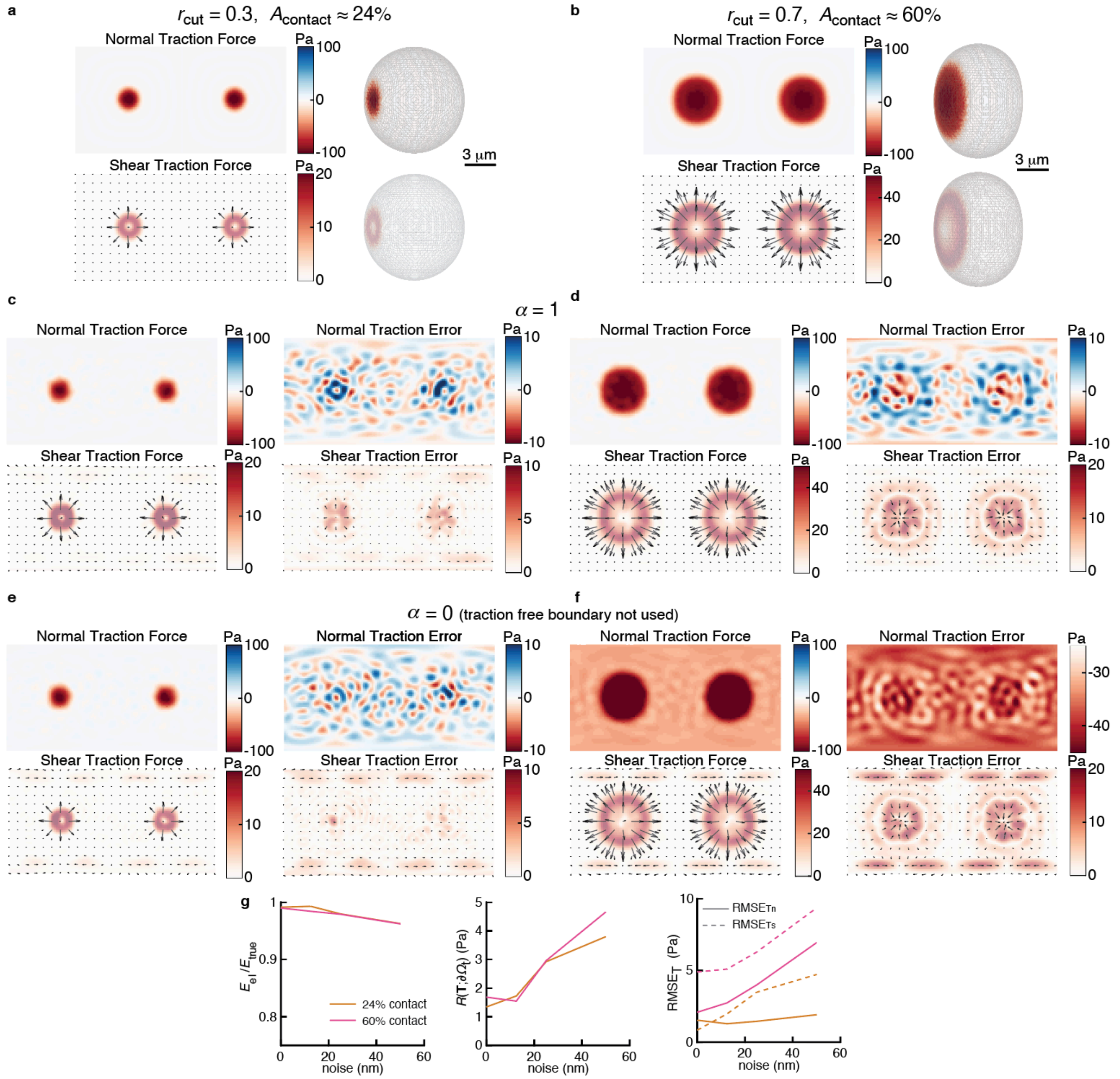

Supplementary Figure 9: Evaluation of the computational strategy with test cases. We designed benchmark test cases with known solutions for testing the accuracy and noise sensitivity of the computational method, as well as for the selection of free parameters α and β. The test case is designed as a sphere with Young's modulus (E_Y) 0.3 kPa and radius 5 μm under a pair of opposing indentations in y-direction. A 100 Pa y-direction traction is applied on the left apex of the sphere and a -100 Pa traction is applied on the right apex. The left-tip region is defined as a surface region $P_{\text{left}} = \{p; p \in \partial\Omega, |p - p_{\text{left}}| < r_{\text{cut}}\}$, similarly the right-tip region $P_{\text{right}} = \{p; p \in \partial\Omega, |p - p_{\text{left}}| < r_{\text{cut}}\}$, where the parameter r_{cut} = 0.1,0.3,…, 0.9 r_0 leads to contact region areas A_{contact} of ~12%, ~24%, ~41%, ~60%, ~84%, respectively, of the sphere surface area $A_{\text{surface}} = 4\pi r_0^2$. Based on this arbitrarily defined traction field as boundary condition, the elastic solution, including the displacement field as well as the deformed shape is completely determined. Importantly, this test case includes a traction-free boundary, and normal as well as significant shear forces. To test the performance of our method in the context of measurement noise, we add random Gaussian noise in the radial direction $r(\theta, \varphi)$ on the deformed shape nodes with standard deviation of 12.5, 25 and 50 nm. For our experimental data, we quantified the noise in radial direction to be ~50 nm pre-smoothing (Fig. 2, main text). Before force analysis, we apply a 2D moving average filter with ~18 points to the shape, which hence results in radial noise of ~12.5 nm. Using the computed deformed shape (with added noise) and the traction free boundary as inputs, we then use our numerical strategy to solve the inverse elasticity problem of recovering the traction forces. Comparing with

the true solution then allows us to evaluate the accuracy of our solution. **a-b,** True solution for r_{cut} = 0.3 (**a**) and 0.7 r_0 (**b**), with both 2D equirectangular map projections of the normal T_n^{true} and shear traction forces $\|\mathbf{T}_s^{true}\|$) (left column) and 3D presentations of the traction forces visualized on the deformed particle shapes (rotated 65º clockwise from front view of the site of force application around the z-axis for visualization). Note that the large-scale indentation in **b** results in considerable expansion along the orthogonal axes. **c-f,** traction fields for the test cases with r_{cut} = 0.3 r_0 (**c,e**) and r_{cut} = 0.7 r_0 (**d,f**) estimated by our reference-free method, with β = 1 and 12.5 nm radial noise. Both computed traction fields (T_n and $\mathbf{T}_s$, left column) and traction errors which indicate the difference of the converged computational solution with the true solution ($T_n - T_n^{true}$ for normal traction, $\|\mathbf{T}_s - \mathbf{T}_s^{true}\|$ for shear traction; right column) are shown. **c-d**, Results with optimal value of weighing coefficient α = 1. Maximum normal traction errors are ~10 Pa in the contact region, which amounts to ~10% of the true traction (~ 100 Pa). Shear traction errors in the contact region are < 8 Pa and < 20 Pa for r_{cut} = 0.3 r_0 and r_{cut} = 0.7 r_0, respectively. This maximum error amounts to approximately 40% of the true shear tractions (~20 Pa & ~ 50 Pa, respectively). Root mean-square traction errors $RMSE_T$ are ~1 Pa for normal tractions ($RMSE_{Tn}$) and ~3 Pa for shear forces ($RMSE_{Ts}$). Residual tractions on the traction-free region are low at $R(\mathbf{T}; \partial\Omega_t) \approx 1.5$ Pa. Overall, the pattern, direction and magnitude of the true forces are captured closely. **e-f,** Results with weighing coefficient α = 0, which corresponds to the solution of the elasticity problem without using a traction free-region. Maximum normal traction errors in the contact region are ~10 Pa and ~50 Pa for r_{cut} = 0.3 r_0 and r_{cut} = 0.7 r_0. Shear traction errors in the contact region are comparable to the solution with α = 1. There are, especially in the case with r_{cut} = 0.7 r_0, significant residual tractions on the traction-free region, in both shear and normal direction, with $(R(\mathbf{T}; \partial\Omega_t)) \approx 2$ Pa and ≈ 30 Pa, for r_{cut} = 0.3 r_0 and r_{cut} = 0.7 r_0 respectively. Overall, the pattern of the true forces is still captured. However, large shear and normal tractions can occur outside the contact area, which also affect the estimated magnitude of forces in the contact area, and this becomes increasingly problematic with large-scale deformations. Moreover, it seems that including the traction boundary condition helps us to avoid local energy minima (such as the solution for r_{cut} = 0.7 r_0). **g,** converged E_{el}, $R(\mathbf{T}; \partial\Omega_t)$, and root mean-square traction error $RMSE_{Tn}$ and $RMSE_{Ts}$ as functions of noise level for r_{cut} = 0.3 r_0 and r_{cut} = 0.7 r_0 cases, with $\alpha = \beta = 1$. The errors in both normal and shear traction field increases approximately linearly with the noise level, and are, even at high noise levels, well below 10 Pa.

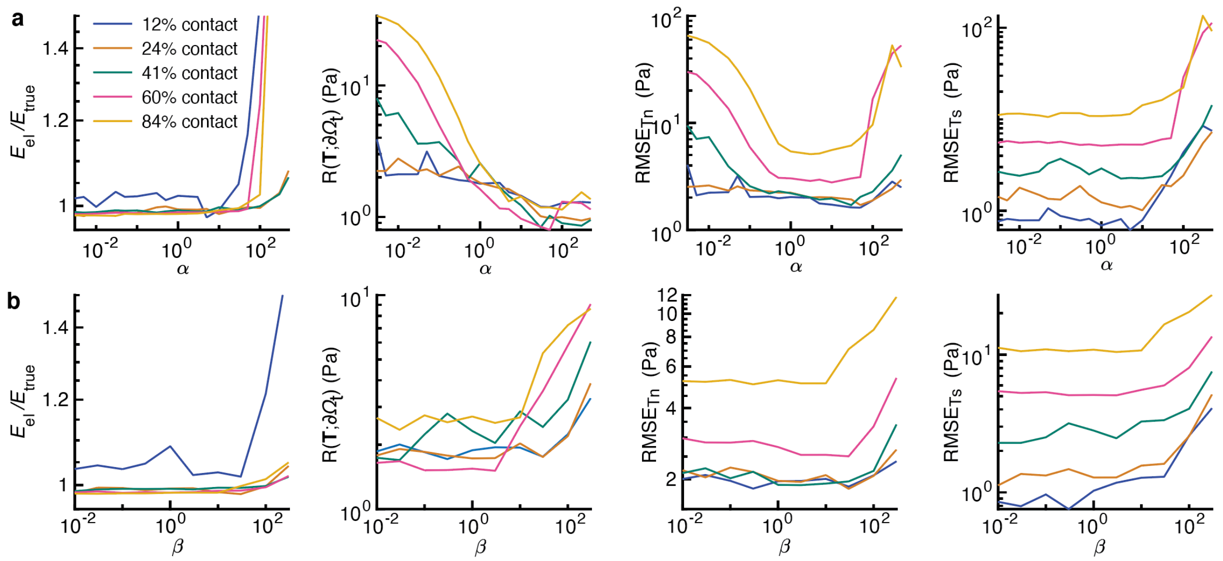

Supplementary Figure 10: Determining free parameters α and β for solving the mixed-boundary problem. Elastic energy E_{el}, residual traction $R(\boldsymbol{T}; \partial\Omega_{\mathrm{t}})$ on the traction-free region, and the mean-squared traction error in both normal ($\mathrm{RMSE}_{\mathrm{Tn}}$) and shear direction ($\mathrm{RMSE}_{\mathrm{Ts}}$) as functions of optimization parameters α and β for the benchmark test cases (Supplementary Fig. 9). E_{el} is normalized by the true elastic energy E_{true} of the test case. Note that the dependence on α and β has the same trend for different contact-area test cases (12 - 84% of the total particle surface). This allows the use of a single α and β value for all shapes. **a,** Converged E_{el}, $R(\mathbf{T}; \partial\Omega_{\mathrm{t}})$, and $\mathrm{RMSE}_{\mathrm{T}}$ as functions of α, with $\beta = 0$. α controls emphasis on the traction-free boundary during optimization: the higher the α value, the lower the traction residual will be on the traction-free region. At $\alpha \approx 1{\sim}10$, the traction residual (< 5 Pa) and mean-squared error of traction field (< 10 Pa) are both low and the energy has plateaued at approximately E_{true}. Larger α quickly leads to $E_{\mathrm{el}} > E_{\mathrm{true}}$, and higher normal and shear traction error, whereas reducing α leads to overfitting of the elastic energy ($E_{\mathrm{el}}/E_{\mathrm{true}} < 1$), larger tractions on the traction-free region and notably higher normal traction errors. Therefore, $\alpha \approx 1{\sim}10$ is the optimal choice. **b,** converged E_{el}, $R(\mathbf{T}; \partial\Omega_{\mathrm{t}})$, and $\mathrm{RMSE}_{\mathrm{T}}$ as a function of β, with fixed $\alpha = 1$. β controls the emphasis on anti-aliasing during optimization: the higher the β value, the smoother the traction field will be. For different contact region areas, anti-aliasing does not obviously influence the converged E_{el}, $R(\mathbf{T}; \partial\Omega_{\mathrm{t}})$, and $\mathrm{RMSE}_{\mathrm{T}}$ when $\beta \leq 1$. Therefore, $\beta = 1$ is an optimal choice for the benchmark test cases.

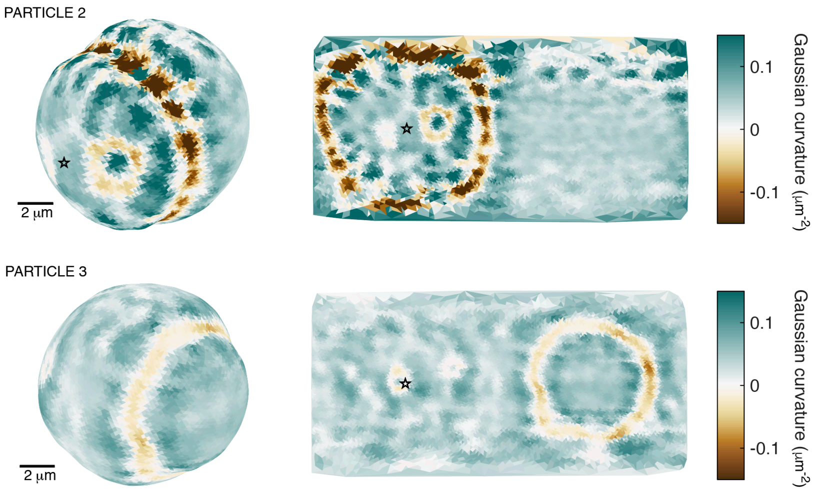

Supplementary Figure 11: Gaussian curvature indicates ring homogeneity differences between particles. A particle in the stage of pseudopod progression (top, 34% engulfed, see particle 2 in main text figure 4b) and a particle in the phase of cup closure (bottom, 84% engulfed, see particle 3 in main text figure 4c) are shown. Gaussian curvature is zero when one of the two principal curvatures is 0, positive when both principal curvatures have the same sign (a bulge or an indentation), and negative when the principal curvatures have opposite signs (a saddle point). This highlights homogeneities in the ring, which are striking in particle 2, but mostly absent in particle 3. Stars mark the phagocytic cup base. Left, projection of particle surface from a perspective that facilitates ring visualization. Right, equirectangular map projections (similar to figure 4 in the main text).

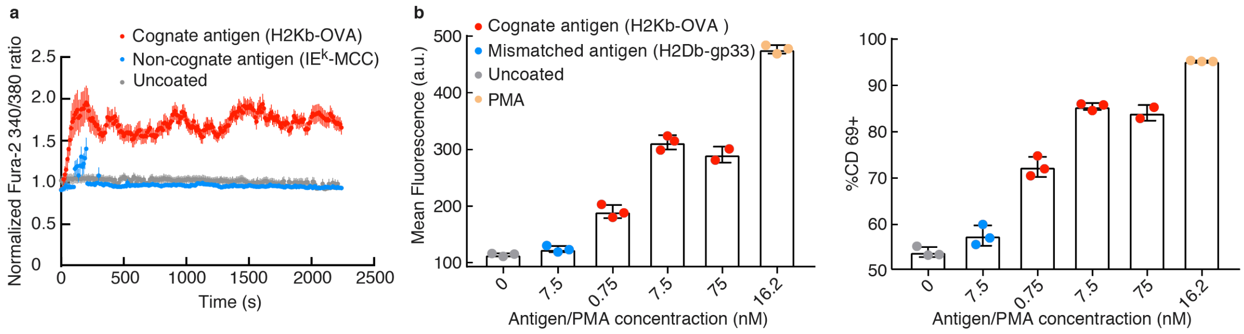

Supplementary Figure 12: OT-I CA^{2+} response and CD69 expression upon incubation with DAAM-particles. **a,** OT-I CA^{2+} response; day 7 CTLs from OT-I transgenic mice were loaded with the intracellular calcium (Ca^{2+}) indicator Fura2-AM and incubated with DAAM-particles (8.6 µm, C_c 2.3%, E_y 4 kPa) coated with either cognate antigen (H2Kb-OVA and ICAM-1, n = 53 events) or non-cognate antigen (MCC-IEk and ICAM-1, n = 81 events), or left uncoated (n = 89 events). Normalized Fura ratios were measured by microscopy and graphed in time. Circles represent mean values and error lines represent standard error of the mean (s.e.m.). Data are representative of at least two independent experiments and are consistent across different stiffness regimes. **b,** T-cell CD69 expression dependence on DAAM-particle coating concentration. 5x10^6 DAAM-particles were incubated with peptide antigens at the given concentrations, and then washed rigorously. For antigens matching the OT-I T-cell receptor, the H2K^b-OVA peptide-MHC complex and ICAM-1 adhesion molecule were used; for the non-cognate (antigen mismatched) condition, H2D^b-gp33 and ICAM-1 were used. (In both cases, 360 nM ICAM-1 was co-coated with the antigens.) Cells were then incubated at 37 °C with DAAMPs for 5 hours, harvested, stained for CD69, and analysed by flow cytometry. Unstained cells and cells stained with an isotype control antibody were used as references for determining voltage settings. For determining the baseline CD69 signal, cells were incubated with uncoated beads (grey); for the maximum potential CD69 signal, phorbol myristate acetate (PMA) was incubated directly with cells (yellow). CD69 expression levels on a per-cell basis (left, as quantified by the geometric mean fluorescence), as well as the proportion of CD69-expressing cells in the population (right), both saturate at a peptide concentration of 7.5 nM. This coating concentration was then used for the Calcium flux and high-resolution imaging experiments. Source data are provided as a Source Data file.

Supplementary Table 1: Concentrations used for conjugation of the MPs

Particle	C_c % (m/m)	*C* BSA (mg/mL)	*C* Ab1 (mg/mL)	*C* Ab2 (µg/mL)	*C* dye mM	Figure
**Intraparticle homogeneity illustration**						
DAAM	2.3	5*	-	-	2.32	1e
DAAM	0.32	5*	-	-	0.58	S2
**Lensing effect illustration**						
DAAM	0.32	19	0.09	4.5	0.58§	1i, S5
PS	-	0.04†	0.06	3	-	1i, S5
**Mechanical characterization/Determination resolution/Sphericity measurements**						
DAAM	2.3	77	-	-	2.32	1j, 1k, S3, S6
DAAM	0.65	29	-	-	0.87	1k, S3, S6
DAAM	0.32	19	-	-	0.58	1k, 2, S6
**Measurements phagocytic efficiency**						
DAAM	2.3	77	-	-	2.32	S8
DAAM	2.3	77	0.14	-	2.32	S8
DAAM	0.65	29	0.11	-	0.87	S8
DAAM	0.32	19	0.09	-	0.58	S8
**Phagocytic deformation assay**						
DAAM	0.32	19	0.09	4‡	0.58	3, 4, 5, S11

Concentration of crosslinker (C_c), BSA, primary antibody (anti-BSA rabbit IgG), secondary antibody (anti-rabbit IgG) used for the various experiments. For all conjugations of deformable acrylamide-co-acrylic acid microparticles (DAAM-particles), the particle concentration was kept at 5% v/v. For polystyrene microparticles (PS MPs) particle concentration was 2% (v/v). BSA concentration was chosen such that enough BSA was present to bind 25% of the acrylic acid groups within the particles. *FITC-BSA instead of BSA was used for derivation, in which case a smaller amount of protein was used. †For polystyrene particles a much lower concentration was used, since the protein cannot diffuse into the non-porous particle. In this case, 10x the amount to saturate the surface area of the particles was used. Primary antibody concentration was chosen such that enough IgG was present for opsonizing 2x the surface of the particles (although the primary antibody may diffuse into the particles). Secondary antibody concentration was picked such that enough antibody was present for ~0.1x surface saturation. ‡Immunostaining was performed after exposure of the beads to cells. Concentration of the carboxyl-reactive dye (TRITC-Cadaverine (Cad)) was chosen such that enough dye was present to bind 50% of the acrylic acid groups within the particles. §Alexa Fluor 488-Cad was used instead of TRITC-Cad. Most right column indicates the figures for which each of these particles was used, where S indicates reference to supplementary figures.

Supplementary Note 1: Spherical harmonic decomposition of particle shape

For determining if there is a characteristic length scale of the deviations of non-deformed particles from a perfect sphere, the edge coordinates of adherent particles (those with the highest signal-to-noise ratio in figure 2a in the main text) were represented in Cartesian coordinates $(x_i, y_i, z_i), i = 1,2, \dots, n$. First, we determine the center (X, Y, Z) and the radius R of the particle by minimizing the sum of the squared distances between the data points to the spherical surface given by (X, Y, Z, R):

$$\min_{(X,Y,Z,R)} \sum_i [(x_i - X)^2 + (y_i - Y)^2 + (z_i - Z)^2 - R^2] \ (1)$$

The data points were then represented in spherical coordinates:

$$r_i = \sqrt{(x_i - X)^2 + (y_i - Y)^2 + (z_i - Z)^2}, \theta_i = \arccos\frac{z_i - Z}{R}, \varphi_i = \mathrm{atan2}(y_i - Y, x_i - X),$$

which can be treated as the radius of points being a function of co-latitude and longitude $r(\theta, \varphi)$. Considering the measurement error increases with the distance to the equator ($\theta = 90°$), the actual radius is corrected using weighted function $w(\theta)$ described in Supplementary Note 2:

$$\bar{r}_\mathrm{i}(\theta_\mathrm{i}, \varphi_i) = w(\theta_i)(r_i - R) + R. \ (2)$$

Since we only obtained data on the upper half of the particle, we copy and reverse the data points to form a complete sphere: $\bar{r}(\theta > 90°, \varphi) = \bar{r}(180° - \theta, \varphi + \varphi_0)$, where φ_0 is a random angle between $(0°, 90°)$ to avoid the symmetry of the upper and lower sphere. Next, using SHTools[4] spherical harmonic analysis was performed on $\bar{r}(\theta, \varphi)$ using the least-square method:

$$\bar{r}(\theta, \varphi) = \sum_{l=0}^{l_{\max}} \sum_{m=-l}^{+l} \hat{r}_l^m Y_l^m(\theta, \varphi), \ (3)$$

where $\hat{r}_l^m$ are the spherical harmonic coefficients, and Y_l^m are the spherical harmonic functions defined in Supplementary Note 5. In the following analysis, the cut-off $l_{\max} = 20$ was used to be consistent with the spherical harmonic method we use throughout the manuscript. Finally, the "per-l" power spectrum of the spherical harmonic coefficients are defined as

$$S_l = \sum_{m=-l}^{+l} (\hat{r}_l^m)^2, (4)$$

for presenting the magnitude of the coefficients as a function of spherical harmonic degree l.

Supplementary Note 2: Gauss-Legendre quadratic (GLQ) meshing, latitude and resolution weighing function

Gauss-Legendre quadratic mesh with degree L is exact if the sampling function $f(\theta, \varphi)$ is terminated within L degrees of spherical harmonics. The longitude nodes are uniform on longitude with $360°/(2L+1)$, and the latitude nodes correspond to zeros of the Legendre Polynomial of degree $l = L + 1$:

$$P_l(\mu) = \frac{1}{2^l l!} \frac{d^l}{d\mu^l} (\mu^2 - 1)^l, (5)$$

where $\mu = \cos\theta$. In total, GLQ has $n_{\mathrm{node}} = (L+1) \times (2L+1)$ nodes on (θ, φ) space. Since GLQ meshing is regular meshing on θ, there will be a higher density of mesh nodes at higher latitude and less nodes at the equator. To compensate for the over-sampling at high latitude, we introduce a weight function $w(\theta) = \sin\theta$, which is applied in the traction residual $R(\mathbf{T}; \partial\Omega_{\mathrm{t}})$.

Supplementary Note 3: Anti-aliasing to suppress Moiré patterns in traction field

Anti-aliasing is incorporated in the minimization algorithm to reduce Moiré patterns in the traction field. The penalty term in the target function f is defined as:

$$E_{\mathrm{pen}} = \sum_{j=1}^{3} \sum_{l=0}^{l_{\mathrm{max}}} \sum_{m=-l}^{l} \left[w_{\mathrm{p}}(l) \hat{T}_{jlm}\right]^2, (6)$$

where the penalty coefficient is defined as a linear function of spherical harmonic degree l in the range of $(l_{\mathrm{cut}}, l_{\mathrm{max}})$:

$$w_{\mathrm{p}}(l) = \begin{cases} 0, & l \le l_{\mathrm{cut}} \\ \dfrac{l_{\mathrm{max}} - l}{l_{\mathrm{max}} - l_{\mathrm{cut}}}, & l_{\mathrm{cut}} < l \le l_{\mathrm{max}} \end{cases}, (7)$$

In this work, we select $l_{\mathrm{cut}} = l_{\mathrm{max}} - 5 = 15$ for a balance between satisfactory anti-aliasing and accuracy. There is another parameter β controlling the magnitude of the penalty. $\beta = 1$ was selected based on the test cases (Supplementary Fig. 10)

Supplementary Note 4: Dilation of the edge of the traction-free region

For calculation of surface traction forces, the traction-free boundary region was dilated because of 1) lack of knowledge about the exact correspondence of the region of possible cellular force exertion on the target with the area of tight proximity between cell and target (where the fluorescent secondary antibody probe is excluded), 2) the notion that overestimation of the extent of the traction-free surface will likely have a more profound effects on the resultant forces than underestimation of this area. Dilation by 1 μm was performed by excluding points from the traction free surface with great circle distance <1 μm to any point part of the edge of the traction-free boundary.

Supplementary Note 5: Spherical Harmonics definition

Complex spherical harmonic functions in this paper is defined as following:

$$Y_l^m(\theta,\varphi) = \begin{cases} \bar{P}_l^m(\cos\theta)\, e^{im\varphi}, m \geq 0 \\ (-1)^{|m|}\left[Y_l^{|m|}\right]^*, m < 0 \end{cases}, (8)$$

where $\left[Y_l^{|m|}\right]^*$ is the complex conjugate of $Y_l^{|m|}$, and $P_l^m(\mu)$ is the normalized associated Legendre function with the complex 4π-normalization which is defined as following:

$$\bar{P}_l^m(\mu) = \sqrt{(2l+1)\frac{(l-m)!}{(l+m)!}} P_{lm}(\mu). (9)$$

The unnormalized associated Legendre functions are defined as:

$$P_{lm}(\mu) = (1-\mu^2)^{\frac{m}{2}} \frac{d^m}{d\mu^m} P_l(\mu), (10)$$

where the Legendre function $P_l(\mu)$ is defined in Supplementary Note 2. Spherical harmonic functions are orthogonal for different (l, m) degrees:

$$\iint_{\partial\Omega} [Y_l^m(\theta,\varphi)]^* \, Y_{l'}^{m'}(\theta,\varphi)\, dS = 4\pi\delta_{ll'}\delta_{mm'}, (11)$$

where $\delta_{ll'}$ is the Kronecker delta function.

Given the orthogonality property of spherical harmonic functions, it is straightforward to evaluate the elastic energy by convolving displacement field and traction field on the spherical surface, which equals to the inner product of spherical harmonic coefficients:

$$E_{\text{el}} = \frac{1}{2}\iint_{\partial\Omega} u_j(\theta,\varphi) \cdot T_j(\theta,\varphi)\, dS = \frac{1}{2}\iint_{\partial\Omega} \left[\hat{u}_{jlm} Y_l^m(\theta,\varphi)\right] \cdot \left[\hat{T}_{jlm} Y_l^m(\theta,\varphi)\right] \mathrm{d}S$$

$$= 2\pi \sum_{j=1}^{3} \sum_{l=0}^{l_{\max}} \sum_{m=-l}^{l} \left[\hat{u}_{jlm}\right]^* \hat{T}_{jlm}, \ (12)$$

where $\left[\hat{u}_{jlm}\right]^*$ is the complex conjugate of the coefficient $\hat{u}_{jlm}$.

SUPPLEMENTARY DISCUSSION

Superresolving the particle boundary by edge localization microscopy (ELM)

In the radial direction, we have been able to resolve the particle boundary with accuracy beyond the diffraction limit (lateral resolution $d_{x,y} \approx 200$ nm, axial resolution $d_z \approx 600$ nm). Such resolution is accomplished similarly to single-molecule localization techniques[5,6]; the particle edge, representing an optically distinct feature, can be localized with precision that increases approximately with $1/\sqrt{N}$, where N is the number of photons collected. For a homogeneously labelled particle, the radial intensity profile can be approximated as the convolution of a step function, representing the homogeneous and dense presence of fluorophores, with a Gaussian function representing the emission spot, *i.e.* the point spread function (PSF), of a single fluorophore. The derivative of this convolution is, in fact, a Gaussian itself, localized at the particle edge. Hence, edge superlocalization can be performed by fitting a Gaussian to the derivative of the intensity profile at the particle edge, where the discrete derivative can, for example, be approximated using the 3D Sobel operator[7].

Synchronized particle polymerization using oil-soluble polymerization initiators

The particle-to-particle variation we observe in optical and mechanical properties is remarkably low. For MPs with a refractive index ~1.4, previously a spread on the order of 0.001 was reported[2], while we observe a ~10 fold lower spread of ~0.0001. This homogeneity, which is also present in mechanical properties (CV = 0.08 - 0.30 for stiff to soft particles), may be related to our choice for an oil-soluble polymerizing agent. Water-soluble initiators, such as ammonium persulfate (APS) have frequently been used for polyacrylamide polymerization, and were also used previously for particle synthesis. Such initiators must be present in the gel mixture during emulsification, since they are not oil soluble. It was previously shown that use of such initiators can lead to differential particle properties (optical and mechanical) over the extrusion period.[2] This could be attributed to polymerization of the gel mixture before extrusion, which occurs, albeit slowly without a catalyst such as tetramethylethylenediamine (TEMED), over the relatively long extrusion period ($\gtrsim$1 h).[2] Another potential explanation is based on the rapid degradation of APS in aqueous solutions, which could result in a higher concentration of APS and more efficient polymerization of droplets extruded early on, while droplets extruded later will polymerize less

efficiently and will therefore have different properties. Regardless of the exact mechanisms that underlie the particle property dependence on extrusion time, use of an oil-soluble initiator (*e.g.* 2,2'-Azobisisobutyronitrile, AIBN) prevents such problems as it prevents polymerization of the gel mixture before extrusion and initiator degradation, and allows triggering polymerization in an entirely synchronized fashion. Using an oil soluble initiator therefore likely improves particle-to-particle homogeneity. Finally, the use of AIBN did not seem to affect the uniformity of the particle polymer network, as evidenced from the homogeneous radial fluorescent staining of the particles (Fig. 1e, Supplementary Fig. 2), which indicates that AIBN diffusion into the microdroplets did not result in any radial particle inhomogeneities.

Choice of experimental and computational strategy for reconstructing traction forces

Traction force microscopy (TFM) is by now a widely used technique in mechanobiology[8]. The classical approach in TFM is the measurement of the displacement of tracer particles embedded in the deformable hydrogel. Various computational approaches exist to reconstruct forces from such displacement fields, including direct TFM (dTFM) in which the stress is directly inferred from strain calculated from image data, and various variants of inverse TFM (iTFM), which often are based on the use of Green's function, finite element based methods (FEM TFM) and adjoint methods. The benefits and disadvantages of these various approaches have been recently discussed in depth[9,10]. The direct approach was recently introduced to deformable microparticles[11]. dTFM has some key advantages, such as its computational efficiency, and suitability for non-linear hydrogel material properties and complex geometries. Nevertheless, in planar TFM, inverse strategies are more commonly taken, because of the high noise-sensitivity and hence quality of data required for dTFM, as well as that forces in dTFM are usually measured away from the true surface which affects the measurement sensitivity. Generally, the choice of the computational approach is closely linked to experimental details, such as the complexity of the geometry of the cell-hydrogel interface, the material properties of the deformable hydrogel, and the resolution with which the data is acquired.

Here, we chose to deviate from the tracer-based methodologies, largely because this renders the method reference-free, which simplifies the approach significantly. Particularly for microparticle-based TFM (MP-TFM), obtaining an undeformed reference-image either before or after measuring the deformed shape can be challenging, since particles can move away or rotate.

In addition to this experimental advantage, there are other potential benefits in terms of sensitivity, such as that sampling of the shape is not limited to the tracer-to-tracer spacing (typically > 1 μm), therefore allowing evaluation of deformation at more closely spaced measurement points. Furthermore, the magnitude of deformation within an elastic material is highest at the site of application of the stress and scales with ~1/*r*, where *r* is the distance to the point of stress application. Thus, measuring stresses directly at the particle boundary likely enhances sensitivity to small forces. Finally, in the recent direct MP-TFM methodology[11], and more generally in dTFM[9], the stress field in the elastic gel is multiplied with its surface normals. Lacking precise knowledge of the location of the surface, it was previously assumed that the particle shape was convex[11]. However, in both biological contexts studied here, we observed many concave surface regions (*i.e.* areas with mean curvature H < 0, main text Fig. 4 & 6), which are hence critical for an accurate description of exerted forces by the cell.

The main disadvantage of our methodology is that the displacement field is not obtained directly, and hence needs to be estimated from deformed shape alone. This makes the approach computationally more demanding than most tracer-based methods. Currently, our computational method leads to convergence in approximately 2 hours. Although computations for many particles can be run in parallel, this still requires more computational power and time than other approaches. When using larger particles, to obtain similar sampling point density, the maximum degree $l_{\max}$ of the spherical harmonic functions Y_l^m (Supplementary Note 5) needs to be increased, which could drastically increase computational time. In addition, while we could accurately reconstruct shear forces in test-cases (Supplementary Fig. 9), shear forces are inferred less directly than in tracer-based traction force microscopy, and may therefore be less accurate. Like most inverse, but not direct, TFM methods[10], our computational approach is only appropriate for small strains ($\varepsilon \lesssim 0.1$) and linear elastic materials, where the latter constraint is a fundamental limitation of the method. Finally, the approach taken here is appropriate for spherical particles, and can likely be extended to other spheroids, but particles of more complex shape might require direct, FEM or adjoint methods.

Together, the strategy taken here is likely most adapt for when using small (< 20 μm) particles and when the accuracy to measure small-scale forces is crucial. A more quantitative comparison, either computational or experimentally, between tracer-based and shape-based MP-TFM could be highly interesting. Moreover, a hybrid technology could be of significant interest.

Although experimentally the most demanding, knowing the precise surface location (and hence surface normals) and complementing the tracer-based data with the more densely sampled surface-based information could enhance the sensitivity and accuracy of tracer-based MP-TFM. On the other hand, addition of tracer-based measurements could probably significantly reduce computational time in shape-based TFM by starting minimization from a more accurate initial estimate, and could perhaps also lead to more accurate shear measurements.

Supplementary References

1. Bonnet-Gonnet, C., Belloni, L. & Cabane, B. Osmotic Pressure of Latex Dispersions. *Langmuir* **10**, 4012–4021 (1994).
2. Girardo, S. *et al.* Standardized microgel beads as elastic cell mechanical probes. *J. Mater. Chem. B* **6**, 6245–6261 (2018).
3. Lankton, S. & Tannenbaum, A. Localizing region-based active contours. *IEEE Trans. image Process.* **17**, 2029–2039 (2008).
4. Wieczorek, M. A. & Meschede, M. SHTools: Tools for Working with Spherical Harmonics. *Geochemistry, Geophys. Geosystems* **19**, 2574–2592 (2018).
5. Betzig, E. *et al.* Imaging Intracellular Fluorescent Proteins at Nanometer Resolution. *Science.* **313**, 1642–1645 (2006).
6. Rust, M. J., Bates, M. & Zhuang, X. Sub-diffraction-limit imaging by stochastic optical reconstruction microscopy (STORM). *Nat. Methods* **3**, 793–795 (2006).
7. Engel, K., Hadwiger, M., Kniss, J. M., Rezk-Salama, C. & Weiskopf, D. Convolution Filtering for Gradient Estimation. in *Real-Time Volume Graphics* 112–113 (A K Peters, Ltd., 2006).
8. Style, R. W. *et al.* Traction force microscopy in physics and biology. *Soft Matter* **10**, 4047 (2014).
9. Legant, W. Measurement of Cellular Forces via Traction Force Microscopy. in *Handbook of Imaging in Biological Mechanics* 291–298 (CRC Press, 2014). doi:10.1201/b17566-31
10. Schwarz, U. S. & Soiné, J. R. D. Traction force microscopy on soft elastic substrates: A guide to recent computational advances. *Biochim. Biophys. Acta - Mol. Cell Res.* **1853**, 3095–3104 (2015).
11. Mohagheghian, E. *et al.* Quantifying compressive forces between living cell layers and within tissues using elastic round microgels. *Nat. Commun.* **9**, 1878 (2018).
