# Supplementary material for: Microparticle traction force microscopy reveals subcellular force exertion patterns in immune cell–target interactions
Source: Nat Commun. 2020 Jan 7;11:20. doi: 10.1038/s41467-019-13804-z (PMC6946705; doi:10.1038/s41467-019-13804-z)
Supplement: Supplementary file 3 — Description of Additional Supplementary Files [file 41467_2019_13804_MOESM3_ESM.pdf]

Description of Additional Supplementary Files

File Name: Supplementary Movie 1
Description: Shape, mean curvature, surface accessibility, and normal and shear traction force magnitude for particle 1 (9 % engulfed).

File Name: Supplementary Movie 2
Description: Shape, mean curvature, surface accessibility, and normal and shear traction force magnitude for particle 2 (34 % engulfed).

File Name: Supplementary Movie 3
Description: Shape, mean curvature, surface accessibility, and normal and shear traction force magnitude for particle 3 (84 % engulfed).

File Name: Supplementary Movie 4
Description: Shape, mean curvature, surface accessibility, and normal and shear traction force magnitude for particle 4 (100 % engulfed).
