# Supplementary material for: Microparticle traction force microscopy reveals subcellular force exertion patterns in immune cell–target interactions
Source: Nat Commun. 2020 Jan 7;11:20. doi: 10.1038/s41467-019-13804-z (PMC6946705; doi:10.1038/s41467-019-13804-z)
Supplement: Supplementary file 8 — Reporting Summary [file 41467_2019_13804_MOESM8_ESM.pdf]

nature research

Corresponding author(s): Julie A. Theriot

Last updated by author(s): Oct 24, 2019

Reporting Summary

Nature Research wishes to improve the reproducibility of the work that we publish. This form provides structure for consistency and transparency in reporting. For further information on Nature Research policies, see Authors & Referees and the Editorial Policy Checklist.

Statistics

For all statistical analyses, confirm that the following items are present in the figure legend, table legend, main text, or Methods section.

n/a	Confirmed	
☐	☒	The exact sample size (n) for each experimental group/condition, given as a discrete number and unit of measurement
☐	☒	A statement on whether measurements were taken from distinct samples or whether the same sample was measured repeatedly
☒	☐	The statistical test(s) used AND whether they are one- or two-sided<br>*Only common tests should be described solely by name; describe more complex techniques in the Methods section.*
☒	☐	A description of all covariates tested
☒	☐	A description of any assumptions or corrections, such as tests of normality and adjustment for multiple comparisons
☐	☒	A full description of the statistical parameters including central tendency (e.g. means) or other basic estimates (e.g. regression coefficient) AND variation (e.g. standard deviation) or associated estimates of uncertainty (e.g. confidence intervals)
☒	☐	For null hypothesis testing, the test statistic (e.g. F, t, r) with confidence intervals, effect sizes, degrees of freedom and P value noted<br>*Give P values as exact values whenever suitable.*
☒	☐	For Bayesian analysis, information on the choice of priors and Markov chain Monte Carlo settings
☒	☐	For hierarchical and complex designs, identification of the appropriate level for tests and full reporting of outcomes
☒	☐	Estimates of effect sizes (e.g. Cohen's d, Pearson's r), indicating how they were calculated

Our web collection on statistics for biologists contains articles on many of the points above.

Software and code

Policy information about availability of computer code

Data collection	Micromanager (v.1.4.23) was used for fluorescence microscopy image experiments, except for the T cell experiments for which Metamorph (v.7.8.13.0) was used. For atomic force microscopy (AFM) experiments Bruker NanoScope (9.10) software was used. Refractive index measurements were made using the software Steve.
Data analysis	Particle shape analysis was performed using custom code in Matlab, which is available in a community repository (https://gitlab.com/dvorselen/DAAMparticle_Shape_Analysis). Force analysis was performed using custom Python code, which is also available in a community repository (https://gitlab.com/micronano/ShElastic). Particle size and refractive index measurements were analyzed using ImageJ (v2.0.0.-rc-69/1.52i). For AFM analysis custom Maltab software was used, which is available from the authors upon request.

For manuscripts utilizing custom algorithms or software that are central to the research but not yet described in published literature, software must be made available to editors/reviewers. We strongly encourage code deposition in a community repository (e.g. GitHub). See the Nature Research guidelines for submitting code & software for further information.

Data

Policy information about availability of data

All manuscripts must include a data availability statement. This statement should provide the following information, where applicable:

- Accession codes, unique identifiers, or web links for publicly available datasets
- A list of figures that have associated raw data
- A description of any restrictions on data availability

The authors declare that the data supporting the findings of this study are available within the article (and its Supplementary Information) or available from the authors upon request.

Field-specific reporting

Please select the one below that is the best fit for your research. If you are not sure, read the appropriate sections before making your selection.

☒ Life sciences ☐ Behavioural & social sciences ☐ Ecological, evolutionary & environmental sciences

For a reference copy of the document with all sections, see nature.com/documents/nr-reporting-summary-flat.pdf

Life sciences study design

All studies must disclose on these points even when the disclosure is negative.

Sample size	No relevant statistical analysis involving comparisons between samples were made, since the manuscript focuses on the description and illustration of the potential of a new methodology.
Data exclusions	No data exclusions were made.
Replication	All attempts at replication were successful.
Randomization	Cells were randomly allocated to experimental groups.
Blinding	Blinding is not applicable to the described experimental designs (i.e. single-cell force measurements of a known cell-type).

Reporting for specific materials, systems and methods

We require information from authors about some types of materials, experimental systems and methods used in many studies. Here, indicate whether each material, system or method listed is relevant to your study. If you are not sure if a list item applies to your research, read the appropriate section before selecting a response.

Materials & experimental systems

n/a	Involved in the study	
☐	☒	Antibodies
☐	☒	Eukaryotic cell lines
☒	☐	Palaeontology
☐	☒	Animals and other organisms
☒	☐	Human research participants
☒	☐	Clinical data

Methods

n/a	Involved in the study	
☒	☐	ChIP-seq
☒	☐	Flow cytometry
☒	☐	MRI-based neuroimaging

Antibodies

Antibodies used	anti-BSA rabbit IgG (MP Biomedicals, 0865111), Alexa Fluor 546 goat anti-rabbit IgG (ThermoFisher Sci., A11035), Alexa Fluor 647 donkey anti-rabbit IgG (ThermoFisher Sci., A31573)
Validation	The only use of primary antibodies was for triggering of macrophage phagocytosis. We validated that J774A.1 macrophage-like cells specifically phagocytosed particles opsonized with this antibody, and not particles functionalized with BSA alone. The relevant validation is described and shown in supplemental figure 8.

Eukaryotic cell lines

Policy information about cell lines

Cell line source(s)	J774A.1 cell lines were obtained from ATCC.
Authentication	None of the cell lines were authenticated.
Mycoplasma contamination	Cell lines were not tested for Mycoplasm contamination.
Commonly misidentified lines (See ICLAC register)	No commonly misidentified cell lines were used.

Animals and other organisms

Policy information about studies involving animals; ARRIVE guidelines recommended for reporting animal research

Laboratory animals	Laboratory mice (M. musculus) used for T-cell experiments were: C57BL/6J (Jackson Laboratory 000664) and OT-I/Rag2 knockout (Taconic 1896). Males and females under 1 year of age were used.
Wild animals	This study did not involve wild animals.
Field-collected samples	This study did not involve field-collected samples.
Ethics oversight	The animal protocols used for this study were approved by the Institutional Animal Care and Use Committee of Memorial Sloan-Kettering Cancer Center.

Note that full information on the approval of the study protocol must also be provided in the manuscript.
